# Three‐domain microbial communities in the gut of *Pachnoda marginata* larvae: A comparative study revealing opposing trends in gut compartments

**DOI:** 10.1111/1758-2229.13324

**Published:** 2024-08-14

**Authors:** Emine Gozde Ozbayram, Sabine Kleinsteuber, Heike Sträuber, Bruna Grosch Schroeder, Ulisses Nunes da Rocha, Felipe Borim Corrêa, Hauke Harms, Marcell Nikolausz

**Affiliations:** ^1^ Department of Marine and Freshwater Resources Management, Faculty of Aquatic Sciences Istanbul University Fatih, Istanbul Turkey; ^2^ Department of Microbial Biotechnology Helmholtz Centre for Environmental Research – UFZ Leipzig Germany; ^3^ Department of Applied Microbial Ecology Helmholtz Centre for Environmental Research – UFZ Leipzig Germany

## Abstract

This study aimed to examine the bacterial, methanogenic archaeal, and eukaryotic community structure in both the midgut and hindgut of *Pachnoda marginata* larvae using an amplicon sequencing approach. The goal was to investigate how various diets and the soil affect the composition of these three‐domain microbial communities within the gut of insect larvae. The results indicated a notable variation in the microbial community composition among the gut compartments. The majority of the bacterial community in the hindgut was composed of Ruminococcaceae and Christensenellaceae. Nocardiaceae, Microbacteriaceae, and Lachnospiraceae were detected in midgut samples from larvae feeding on the leaf diet, whereas Sphingomonadaceae, Rhodobacteraceae, and Promicromonasporaceae dominated the bacterial community of midgut of larvae feeding on the straw diet. The diet was a significant factor that influenced the methanogenic archaeal and eukaryotic community patterns. The methanogenic communities in the two gut compartments significantly differed from each other, with the midgut communities being more similar to those in the soil. A higher diversity of methanogens was observed in the midgut samples of both diets compared to the hindgut. Overall, the microbiota of the hindgut was more host‐specific, while the assembly of the midgut was more influenced by the environmental microorganisms.

## Graphical abstract

The gut microbial community composition of *Pachnoda marginata* larvae nurtured on two diets (leaf vs. straw) was assessed by a three‐domain amplicon sequencing approach. The diet significantly influenced the methanogenic archaeal and eukaryotic community patterns. The hindgut microbiota was more host‐specific, while the community assembly of the midgut was influenced more by the environment.

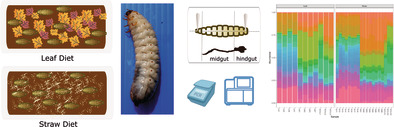

## INTRODUCTION

Within the animal clade, insects have the highest diversity in terms of both the number of species and ecological functions (Engel & Moran, 2013). The soil macrofauna, including earthworms, termites, and insect larvae, among others, plays a crucial role in the carbon cycle as it initiates the decomposition of organic matter (Egert et al., 2003; Takasuka et al., 2013). These animals can consume a variety of organic materials, including nutrient‐poor fibrous lignocellulosic substrates (Ozbayram et al., 2020; Schroeder et al., 2022). Various microorganisms from all three domains, including bacteria, archaea, fungi, and protozoa, colonize their gut and decompose the feed (Arias‐Cordero et al., 2012; Colman et al., 2012; Ebert et al., 2021; Wang et al., 2022). Gut microbiota are considered the fundamental components of insects during their lifecycle (Engel & Moran, 2013), and the mutualistic relationship between the host and the microbiota in the intestinal tract is a key factor enabling the insect to feed on nutritionally poor, recalcitrant diets (Estes et al., 2013; Gontang et al., 2017). The diversity of these microbial communities depends on several factors and is host‐specific, but according to previous studies, it is mainly determined by the host's diet (Colman et al., 2012; Ebert et al., 2021; Yun et al., 2014).

Coleoptera is the largest insect order comprising more than one‐third of insect species (Kergoat et al., 2014), including the Scarabaeidae family (Huang et al., 2010). Many scarab species are known to feed on nutrient‐poor lignocellulose‐rich diets at the larval stage (Huang et al., 2010, 2012). The gut system of *Pachnoda marginata* (Coleoptera: Scarabaeidae) larvae is an excellent example of an efficient micro‐scale biomass conversion system, and the insect hosts are often referred to as “ecosystem engineers” (Ozbayram et al., 2020). The larvae can degrade 65% of the ingested plant fibre material (Cazemier et al., 2003) in their specialized gut system. The gut is composed of three compartments, namely the foregut, the highly alkaline midgut, and the hindgut, referred to as a fermentation chamber (Huang et al., 2010), in which the organic material is processed sequentially and compounds necessary for the host are produced (Schroeder et al., 2022). The digestion starts in the mouth by reducing the particle size of the feed increasing the surface area (Huang et al., 2010). The alkaline environment of the midgut, with pH levels ranging from 9.5 to 11.7 (Cazemier et al., 2003), aids in softening the lignocellulose structure, thus rendering it suitable for cellulolytic activities in the pH‐neutral hindgut (Schroeder et al., 2022). The pH gradient through the gut system enables an efficient digestion and absorption of nutrients (Huang et al., 2010). The gut system hosts a diverse microbial community, which digests lignocellulosic compounds and provides fermentation products that are consumed by the host (Andert et al., 2010; Egert et al., 2003). Prior studies have noted the importance of the hindgut bacteria in cellulose/hemicellulose degradation (Cazemier et al., 2003; Ozbayram et al., 2020), in which the number of bacteria present in the hindgut is known to be 100–1000 times greater than that of the midgut (Cazemier et al., 1997). Furthermore, the specialized microbiota can be transferred to another individual by maternal and/or environmental acquisitions (e.g., feed, soil), and the food source can influence the microbial community structure (Mason et al., 2019). When the imago and the larva have different lifestyles, the gut endosymbionts can be derived from the environment through horizontal transmission. In this strategy, the animals defecate and feed in the same habitat and acquire microorganisms from the environment (Ozbayram et al., 2020). Thus, the soil can have a great impact on the microbial communities of the gut of those insects.

Recent developments and cost reductions in high‐throughput amplicon sequencing as well as advances in data analysis have facilitated studies investigating lignocellulose‐degrading communities (Ozbayram et al., 2020) in various organisms such as Coleoptera imago (Mason et al., 2019) and larvae (Huang et al., 2012; Mohammed et al., 2018; Schroeder et al., 2022). Although some earlier studies assessed the microbial communities in the digestive system of *P. marginata* larvae (Andert et al., 2010; Cazemier et al., 2003; Egert et al., 2003), their methodology did not have the depth to reveal the complexity of the gut microbiome. In addition, most literature focussed on a single group of organisms, mostly bacteria, with limited attention given to a broader ecological context. Since these larvae can survive in lignocellulose‐rich environments, it would be illuminating to understand the interactions between prokaryotic and eukaryotic microorganisms that inhabit their gut systems and their role in breaking down plant matter and nutrient uptake (Mohammed et al., 2018).

This study aimed to explore the bacterial, methanogenic archaeal, and eukaryotic community structure of the midgut and hindgut of *P. marginata* larvae by an amplicon sequencing approach while addressing the question of how different diets induce variations in the composition of three‐domain microbial communities. The investigation also considered the influence of soil microbiota on the gut microbial diversity of insect larvae.

## EXPERIMENTAL PROCEDURES

### Sample preparation

**FIGURE 1:**
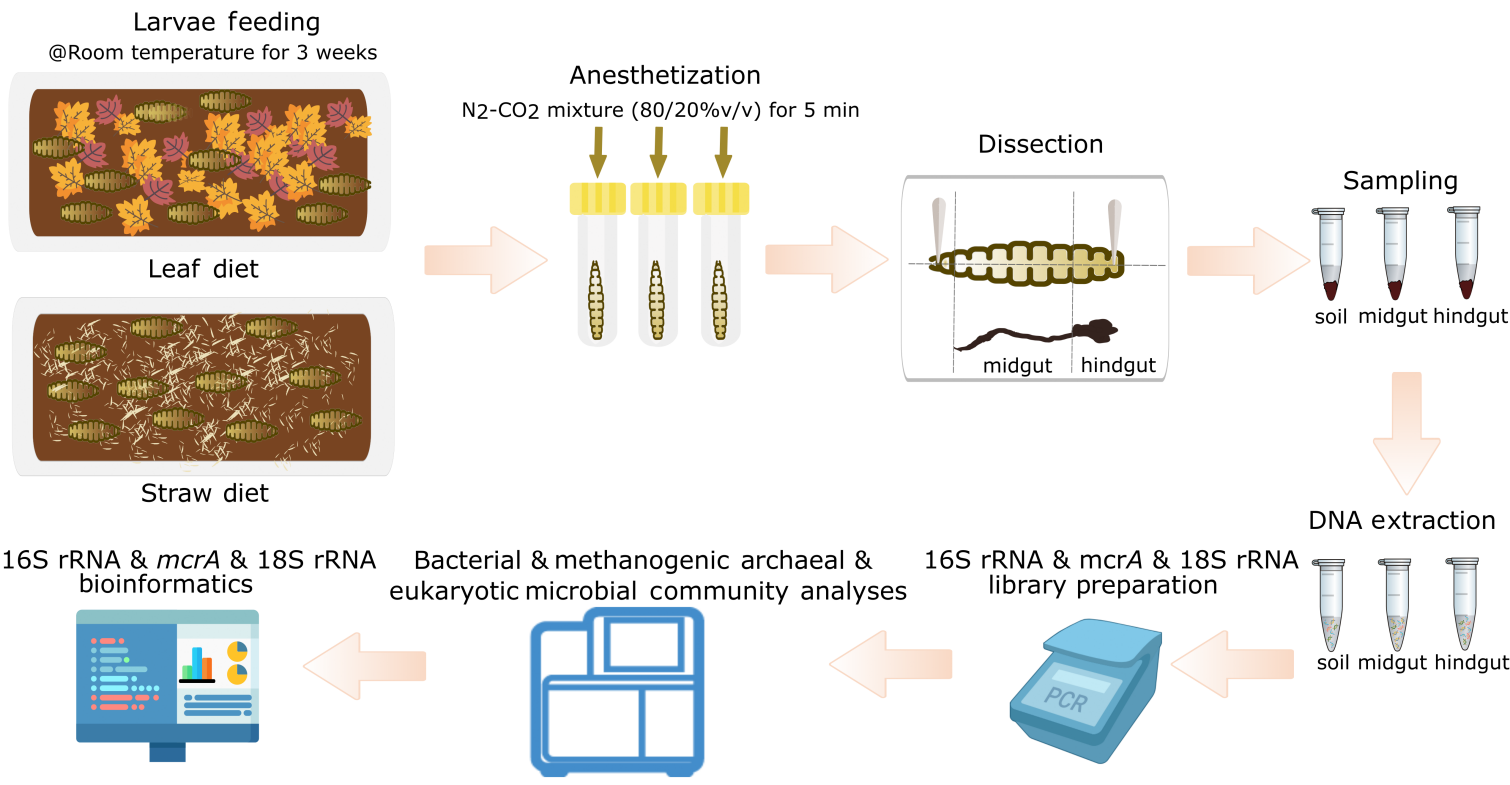
Experimental workflow. Larvae were kept on two distinct diets rich in lignocellulose for 3 weeks at room temperature. Eight individuals were dissected, and samples were taken from their midguts and hindguts, as well as from the soil they inhabited. After DNA extraction, amplicons were prepared with specific primer sets targeting 16S rRNA, 18S rRNA, and *mcrA* genes. Sequencing was done on the Illumina MiSeq platform.

*Pachnoda marginata* larvae were sourced from a commercial breeder (Bugs International GmbH, Irsingen/Unterfeld, Germany). Figure 1 describes the experimental approach used in this study. Larvae were kept on two different diets at room temperature. The first diet was prepared to simulate natural systems, comprising dry leaves, dead wood, and garden soil, while the second diet consisted of only wheat straw in the same garden soil. Twenty individuals were nourished on each diet for 3 weeks. Dissection was carried out according to Lemke et al. (2003) with some modifications. Each individual was placed in a glass vial and anesthetised with an N_2_–CO_2_ mixture (80/20% v/v) for 5 min. Then, eight larvae from each diet were dissected using sterile dissection tools in a laminar flow cabinet to avoid contamination, and midgut and hindgut sections were separated. Care was taken to avoid cross‐contamination of the two gut sections' luminal fluids. The soil samples from each diet were also collected in triplicates. All samples were stored at −20°C until DNA extraction.

### Microbial community analysis

The gut samples were pulverized in liquid nitrogen before DNA extraction. DNA was isolated from homogenates using the NucleoSpin® Soil Kit (Macherey‐Nagel) following the manufacturer's instructions and stored at −20°C until further analysis. The soil samples were also taken and processed in the same way as the gut samples.

The bacterial, eukaryotic, and methanogenic archaeal communities were determined by amplicon sequencing (Illumina MiSeq) of 16S rRNA, 18S rRNA, and *mcrA* genes, respectively, following the procedure described by Feng et al. (2020). The V3–V4 region of the 16S rRNA gene was amplified using the primers 341f (5′CCTACGGGNGGCWGCAG 3′) and 785r (5′GACTACHVGGGTATCTAAKCC3′) (Klindworth et al., 2013). The PhiX Control v3 Library was employed as a control, following the protocol outlined by Illumina. The methanogen‐specific *mcr*A gene was amplified using the primers mlas (GGTGGTGTMGGDTTCACMCARTA) and mcrA‐rev (CGTTCATBGCGTAGTTVGGRTAGT) (Steinberg & Regan, 2008) and amplification of the V8–V9 region of 18S rRNA genes was carried out with the primers 1422f (5′‐ATAACAGGTCTGTGATGCCCT‐3′) and 1797r (5′‐GCCTCCYGCAGGTTCACCTAC‐3′) (Bradley et al., 2016). The raw sequencing data from demultiplexed samples were processed using QIIME2 v2020.2. The DADA2 plugin was used to denoise paired‐end reads, dereplicate, filter out chimeras, and generate amplicon sequence variants (ASVs), following the developer's instructions (Callahan et al., 2016). The sequences were normalized after rarefaction analyses to the lowest read number obtained among the samples. The rarefaction was performed individually for 16S rRNA, 18S rRNA, and mcrA reads. The SILVA database (Version 132) was used to classify the ASVs obtained from 16S and 18S rRNA gene amplicon sequencing (Yilmaz et al., 2014). The taxonomic assignment of *mcr*A ASVs was carried out using a database described earlier (Popp et al., 2017). Estimated marginal means (aka least squares means) of the relative abundances were calculated using the pairwise option with *p*‐value adjustment of Tukey using “emmeans” function in “emmeans” R package (Russell et al., 2023).

Shannon indices were determined using the R package phyloseq (McMurdie & Holmes, 2013), and diversity differences were calculated based on the analysis of variances (ANOVA) with a significance level of *p* < 0.05 in R v. 4.3.0 (R Core Team, 2021). Differences in microbial community compositions (β‐diversity) were calculated using the Bray–Curtis dissimilarity index based on rarefied and square‐root‐transformed ASV abundances, which are visualized as nonmetric multidimensional scaling (NMDS) plots. Permutational multivariate analysis of variance (PERMANOVA) (Anderson, 2017) was calculated by the “adonis2” function in the “vegan” R package using 10^6^ permutations (Oksanen et al., 2020).

Further, we modified a protocol to identify ASVs that could classify the different gut compartments using random forest analysis (Lian et al., 2020; Liu et al., 2022; Rosado et al., 2019), hereafter, bioindicators. Random forest has proven to be superior to other machine learning algorithms in determining bioindicators (Liu et al., 2022). Briefly, we determined if ASV relative abundance could train a random forest model separating different groups of samples (i.e., soil, gut compartments, feed). When groups of samples could be separated (i.e., error rate lower than 5%), we analysed the order of importance of the different ASVs for grouping these samples using the Mean Decrease Gini index for each ASV in the respective random forest models. To select the bioindicators, we first organized the ASVs based on the Mean Decrease Gini in descending order. Thereafter, we created a loop to sum the Mean Decrease Gini index for the different ASVs. We stopped the loop when the next ASV would show a Mean Decrease Gini index smaller than 5% of the sum of the ASVs in the list. We performed these analyses using two inputs: (i) the prokaryotic ASV table and (ii) the prokaryotic and eukaryotic ASV tables concatenated.

## RESULTS

### Bacterial community composition

**FIGURE 2:**
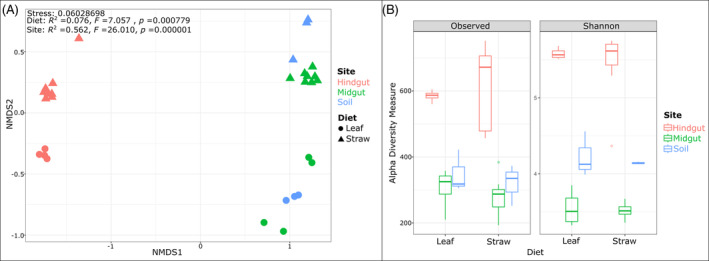
(A) Nonmetric multidimensional scaling (NMDS) analysis and PERMANOVA of the Bray–Curtis dissimilarity index of the bacterial communities based on 16S rRNA gene amplicon sequencing. (B) Observed ASVs and Shannon diversity indices of the bacterial communities.

The bacterial community compositions in the gut compartments and soil were compared for two diets using NMDS analysis (Figure 2A). Bacterial community structures of the hindgut samples were clearly different from those associated with the midgut and soil samples (*p* < 0.05). Furthermore, the diet affected the hindgut bacterial communities (*p* < 0.05), in which two clusters were observed, and the samples obtained from larvae on different diets were clearly separate. The midgut bacterial communities were similar to those in the soils, as the datapoints clustered together. PERMANOVA revealed that both the diet and the compartmentalisation had an impact on shaping the bacterial community patterns of the samples. The bacterial community diversity varied across gut compartments, soil, and feed (Figure 2B). Relatively low diversity was calculated for the midgut samples for both diets, which were similar to the soil samples. While there was a significant difference between the bacterial diversity of the gut compartments (*p* < 0.05), the diversity did not vary significantly with the diets (*p* > 0.05).

The hindgut bacterial communities were dominated by members of Firmicutes (70%–94%, mean: 86.8%), Actinobacteria (3%–15%, mean: 6%), and Proteobacteria (1%–12%, mean: 4%) regardless of the diet (Figure S1). On the other hand, Actinobacteria members dominated the bacterial communities of the midgut samples (mean: 51%). Proteobacteria was found to be the second most abundant phylum (straw‐fed larvae mean: 43%; leaf‐fed larvae mean: 19%). Firmicutes were detected in higher abundances in the midgut samples collected from leaf‐fed larvae (mean: 16%) than in those from straw‐fed larvae (mean: 4%). Bacterial community profiles of the soil where the larvae were kept were similar to the midgut and differed clearly from the hindgut compartments. The soil communities were dominated by Proteobacteria (45%–54%, mean: 48%) and Actinobacteria (35%–45%, mean: 39%). At the class level, Clostridia were most abundant across all the hindgut samples (mean: 86%) (Figure S2). The relative abundances of Actinobacteria and Alphaproteobacteria were higher in the midgut samples (mean: 47% and 35%, respectively) compared to the hindgut bacterial communities (mean: 5% and 4%, respectively) (*p* < 0.0001). Similar to the midgut bacterial communities, the bacterial communities of the soil samples were dominated by Actinobacteria (mean: 35%) and Alphaproteobacteria (mean: 47%) species.

**FIGURE 3:**
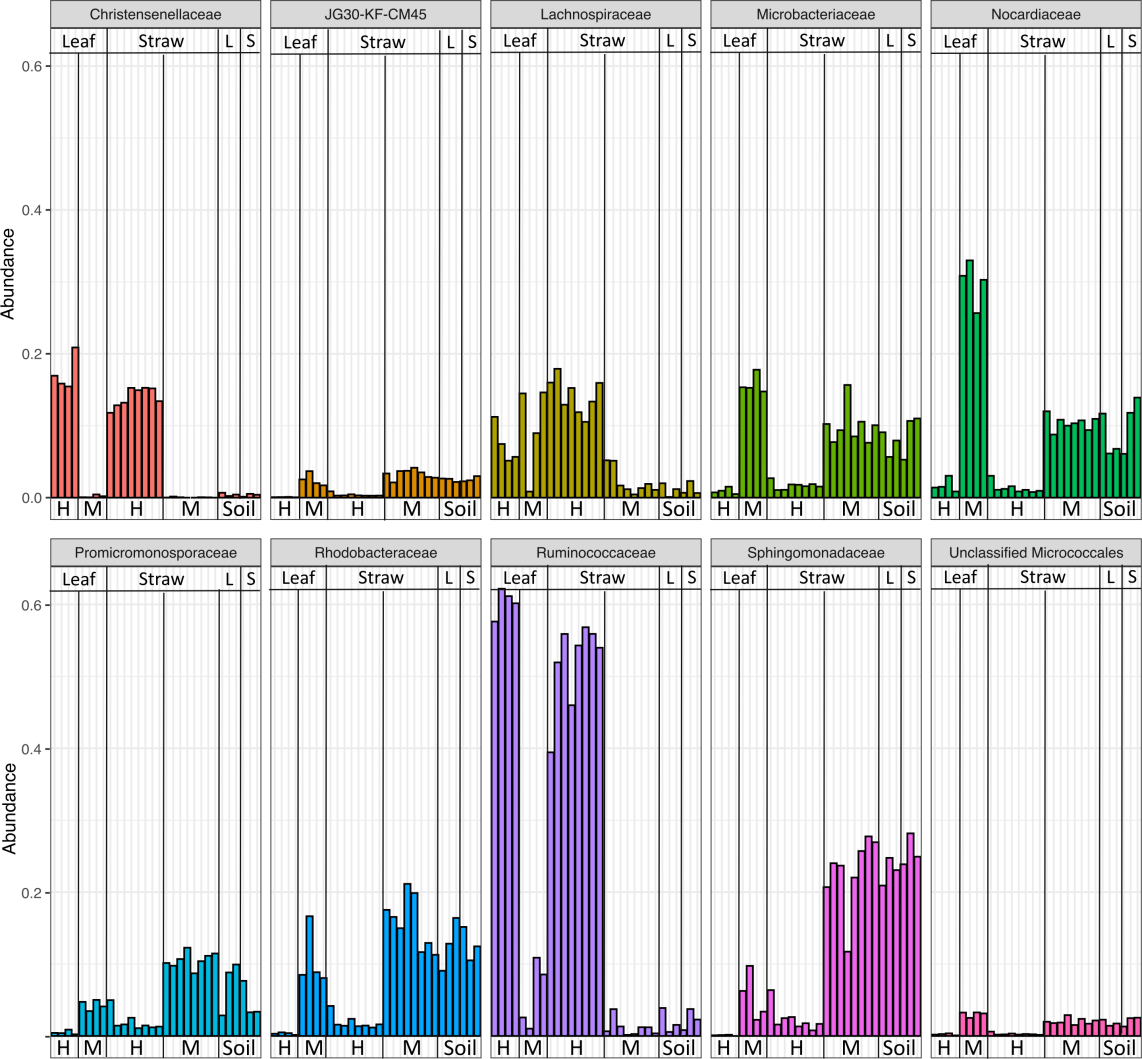
Relative abundance of the 10 most abundant bacterial families in the samples (L: Leaf, S: Straw, H: Hindgut, M: Midgut).

The most abundant bacterial families in the gut microbiota and soil are given in Figure 3. While Ruminococcaceae (mean: 55%), Christensenellaceae (mean: 15%), and Lachnospiraceae (mean: 12%) were found to be the predominant families in the hindgut bacterial communities, the majority of ASVs from soil samples was assigned to the Sphingomonadaceae (mean: 24%), Rhodobacteraceae (mean: 13%), Nocardiaceae (mean: 9%), and Microbacteriaceae (mean: 8%). Although Nocardiaceae (mean: 30%), Microbacteriaceae (mean: 16%), and Lachnospiraceae (mean: 10%) were found to be the major families in the midgut bacterial communities of the larvae with leaf diet, the bacterial communities of the midgut of straw‐fed larvae were dominated by Sphingomonadaceae (mean: 23%) and Rhodobacteraceae (mean: 16%) followed by Promicromonasporaceae (mean: 11%), Nocardiaceae (mean: 10%), and Microbacteriaceae (mean: 10%). The most abundant bacterial genera of the gut compartment and the soil are shown in the supplemental material (Figure S3).

### Methanogenic community composition

**FIGURE 4:**
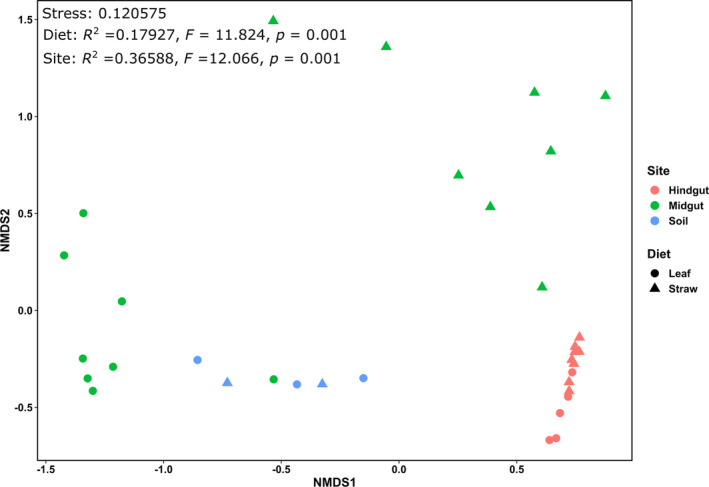
Nonmetric multidimensional scaling (NMDS) analysis and PERMANOVA of the Bray–Curtis dissimilarity indices of the methanogenic archaeal communities based on *mcrA* amplicon sequencing.

NMDS analysis of the methanogenic archaeal communities showed that the two different diets resulted in significant changes in the community structure (*p* < 0.05) (Figure 4). The data points of the hindgut samples generally clustered on one side of the plot and were separated from the midgut and soil samples. On the other hand, the communities in the midgut samples from straw‐fed larvae were different from those from leaf‐fed larvae. The diet was a significant factor that influenced the methanogenic archaeal community patterns of the samples (*p* < 0.05), and the communities were significantly different in the gut compartments and soil (*p* < 0.05).

**FIGURE 5:**
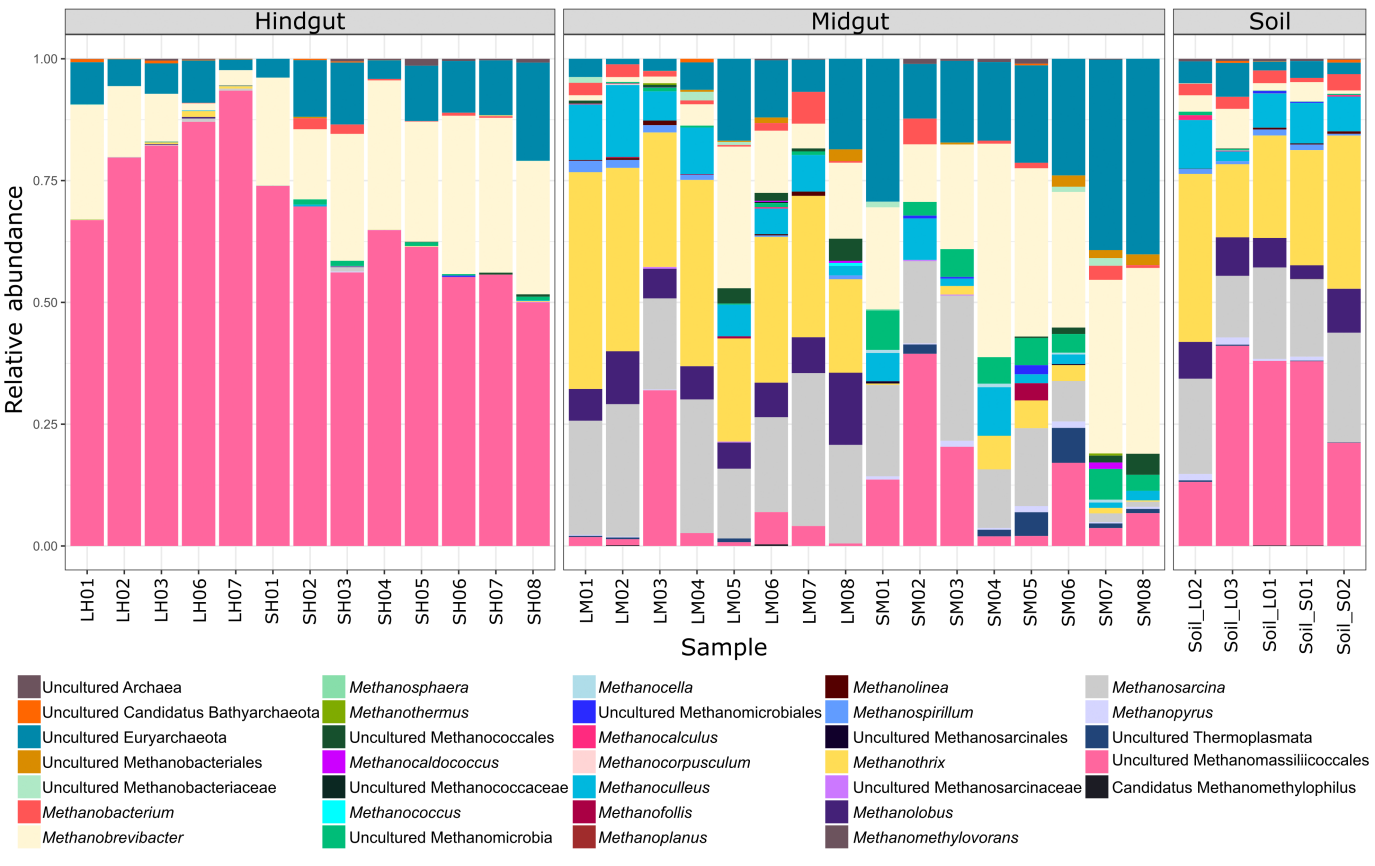
Composition of the methanogenic communities within the larval gut compartments and soil based on *mcrA* gene amplicon sequencing.

The methanogenic communities of the hindgut samples were mainly composed of Methanomassiliicoccales (mean: 69%) and the majority of the reads could not be assigned at the genus level (Figure 5). On the other hand, the communities of the midgut samples were more diverse than those of the hindgut samples. While *Methanothrix* (mean: 31%), *Methanosarcina* (mean: 23%), and *Methanolobus* (mean: 8%) were the dominant genera in the midgut of leaf‐fed larvae, *Methanobrevibacter* (mean: 29%), and *Methanosarcina* (mean: 13%) were abundant in the straw‐fed larval midgut. Additionally, *Methanoculleus* was detected in all midgut microbiota (mean: 6%). *Methanothrix* (mean: 25%), *Methanosarcina* (mean: 18%), and *Methanoculleus* (mean: 7%) and unclassified Methanomassiliicoccales (mean: 30%) contributed to the majority of the soil methanogenic community, but their proportions varied across samples.

### Eukaryotic community composition

**FIGURE 6:**
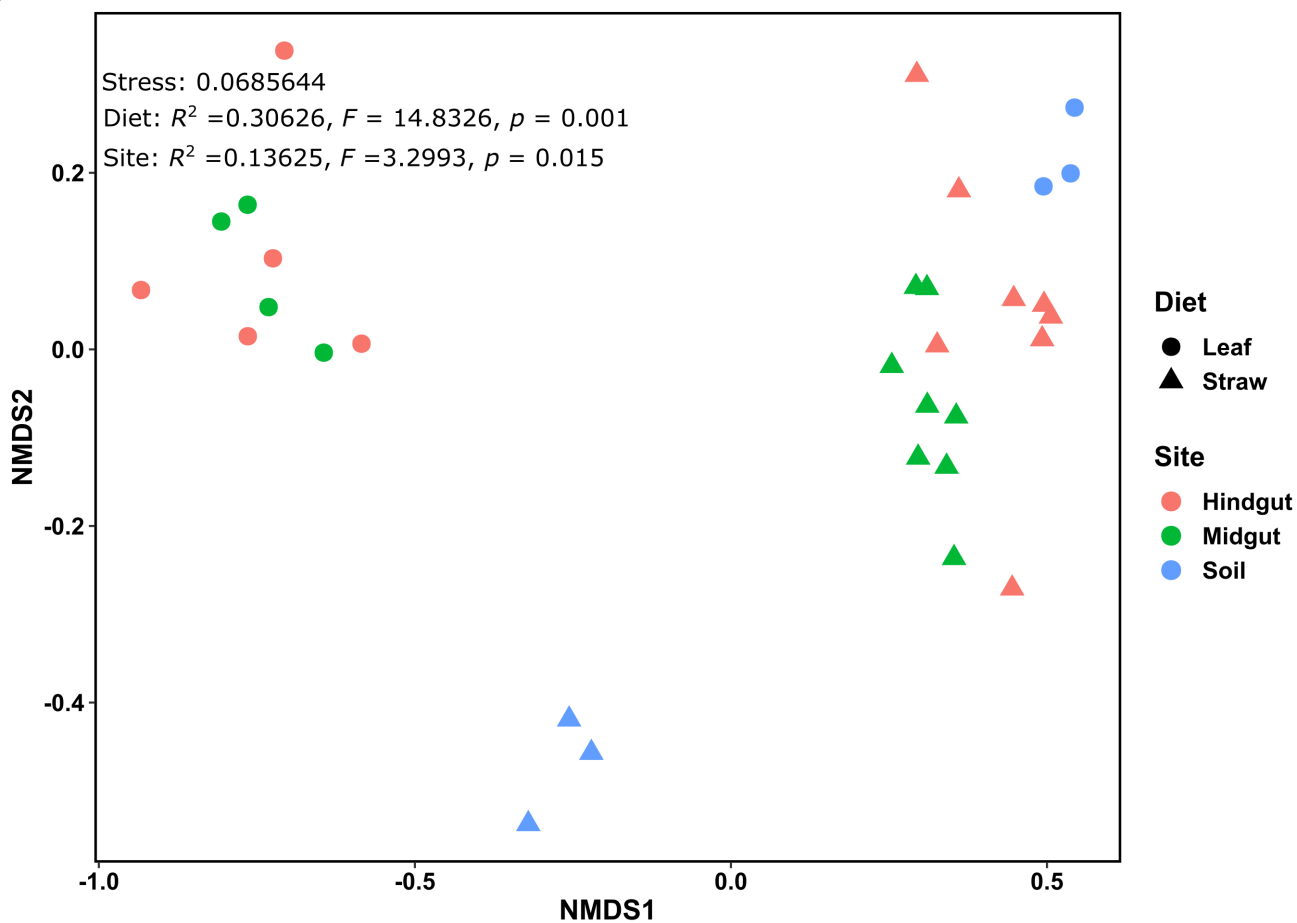
Nonmetric multidimensional scaling (NMDS) analysis and PERMANOVA reflecting the eukaryotic communities in the gut microbiome and soil.

Variations in the eukaryotic communities of the samples were visualized in NMDS plots, which revealed four major clusters (Figure 6). The diet caused a clear shift in the eukaryotic community composition (NMDS analysis, Adonis test; *p* < 0.05). Additionally, analyses showed that the midgut, hindgut, and soil communities were significantly different (*p* < 0.05).

**FIGURE 7:**
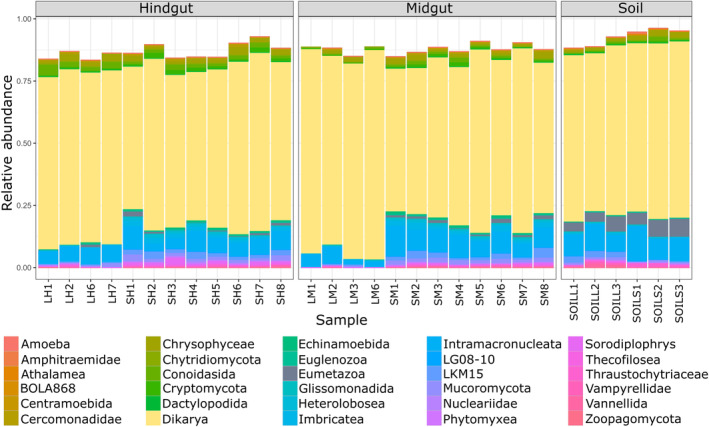
Composition of the eukaryotic communities within larval gut compartments and soil based on 18S rRNA gene amplicon sequencing.

Eukaryotic communities associated with the gut compartments and soil samples are depicted in Figure 7. The predominant eukaryotic order encountered in the samples was Dikarya (Fungi) (mean values for the midgut: 79%, hindgut: 76%, and soil: 73%). It was followed by Intramacronucleata (Chromista) (mean values for the midgut: 8%, hindgut: 7%, and soil: 11%). While Eumetazoa (Animalia) were found at higher abundances in the soil (mean: 6%), Crysophyceae (Chromista) were abundant in the hindgut (mean: 2%). Intramacronucleata were generally found in higher abundances in gut compartments of straw‐fed larvae (mean values for the midgut: 10% and hindgut: 7%) compared to leaf‐fed larvae (mean values for the midgut: 5% and hindgut: 8%).

### Identification of bioindicators

Using ASVs as input for the random forest analyses, we could classify the samples as gut (hindgut + midgut), leaf diet, hindgut of straw diet, midgut of straw diet, and soil. When the input data was in the prokaryotic ASV table, one sample was misclassified (Table S1). From the 18 bioindicators identified using the prokaryotic ASV table as input (Figures S4 and S5), three were assigned to Micrococcales. ASVP2704 assigned to Unclassified Promicromonosporaceae was abundant in both the soil and midgut samples, regardless of the feed. ASVP3601 assigned to Unclassified Lachnospiraceae was found in both hindgut and soil samples, whereas ASVP0902 assigned to Unclassified Rhodospirillales was abundant only in the soil samples. When the input data was the prokaryotic and fungal ASV tables concatenated, no sample was misclassified (Table S1). Out of the 18 bioindicators determined using concatenated input, 11 were prokaryotic ASVs and seven were eukaryotes, of which four were fungal ASVs (Figures S6 and S7). ASVE0879 assigned to Unclassified Debaryomycetaceae and ASVE0802 assigned to Unclassified Pezizomycotina were abundant in all samples. ASVP0638 assigned to Ruminococcaceae UCG‐010 was only found in the hindgut samples. Two bioindicators were assigned to *Candidatus* Soleaferrea, with ASVP1257 being abundant in leaf‐fed larvae and ASVP3674 being abundant in straw‐fed larvae.

## DISCUSSION

This study aimed to explore the effects of different lignocellulose‐rich diets on the microbiomes of gut compartments of *P. marginata* larvae. The results revealed that the microbial community composition in the gut compartments differed significantly. These results seem to be consistent with other studies on *Pachnoda* spp. larvae, which found distinctive patterns of bacterial communities in the midgut and hindgut (Andert et al., 2010; Egert et al., 2003). A study assessed the bacterial communities in the larval gut of *Costelytra zealandica*, another species of Coleoptera (Scarabaeidae), and observed that diet had a significant effect on bacterial community structure in the midgut (Zhang & Jackson, 2008). In contrast, the hindgut community was more stable. In our study, the source of the lignocellulose‐rich diet played a key role in bacterial, methanogenic and eukaryotic community structures. Furthermore, a previous study evaluating the diversity of the gut compartments of *P. marginata* revealed noticeably lower diversity in the midgut than in the hindgut (Andert et al., 2010), which is consistent with our results.

Actinobacteria was the dominant phylum of the bacterial community in the midgut. Since some members of Actinobacteria can sustain metabolic functions under aerobic conditions, a possible explanation for the high abundance of this phylum in the midgut is that due to the tubular structure of this compartment, more oxygen can penetrate it compared to the enlarged sack‐like paunch of the hindgut. Aerobic cellulolytic Actinobacteria use soluble cellulases and hemicellulases with one or more carbohydrate‐binding domains (Anderson et al., 2012). A previous study suggested that the bacterial community in the midgut of *P. marginata* larvae lacks cellulolytic activity (Cazemier et al., 2003). However, Egert et al. (2003) remarked that Actinobacteria species may play a role in the degradation of polysaccharides in the midgut of *Pachnoda ephippiata*. We assume that Actinobacteria were involved in the aerobic cellulose degradation in the midgut, but further metatranscriptomic or metaproteomic analyses are needed for clarification. In detail, among the members of Actinobacteria, the midgut harboured Microbacteriaceae, Cellulomonadaceae, and Promicromonosporaceae as the dominant families. Species of these families can degrade different carbohydrates such as glucose, maltose, cellulose, etc. (Rosenberg, 2014; Stackebrandt et al., 2006).

The hindgut is the major compartment for microbial lignocellulose digestion in scarab larvae (Huang et al., 2010). Our findings showed that bacterial communities in the hindgut compartment varied among individuals, thus agreeing well with the observations of Andert et al. (2010) whose T‐RFLP analysis revealed significantly different hindgut bacterial communities in individual *P. marginata* larvae. In our study, most of the ASVs in the hindgut were affiliated with Firmicutes and Proteobacteria, and the majority were assigned to Ruminococcaceae and Christensenellaceae. These results match those of earlier studies (Schroeder et al., 2022; Yun et al., 2014). Scarab larvae, along with termites and cockroaches, are among the best‐known insects that produce methane (Brune, 2018). Most of the hindgut community was composed of Methanomassiliicoccales (Thermoplasmata). Methanomassiliicoccales species are methanogens that rely on hydrogen as an electron source and convert methylamines and methanol into methane (Söllinger et al., 2018). Besides the presence in human faeces (Poulsen et al., 2013), hydrogen/methanol‐utilizing Thermoplasmata were found in cow rumen (Poulsen et al., 2013) and also detected at high abundance in a methanogenic enrichment culture derived from the termite gut (Paul et al., 2012). Our results are in line with other studies suggesting the dominance of hydrogenotrophic methanogenesis in the hindgut (Egert et al., 2003, 2005; Schroeder et al., 2022) and hydrogen‐dependent methylotrophic methanogenesis (Ozbayram et al., 2020). Furthermore, our findings demonstrated that the methanogen diversity in the midgut was higher than that in the hindgut, which is opposite to the trend observed in the bacterial diversity.

While the midgut of the leaf‐fed larvae community was dominated by members of the acetoclastic methanogens *Methanothrix* and *Methanosarcina*, both hydrogenotrophic and acetoclastic methanogens were abundant in the straw‐fed larval midgut. *Methanothrix* members were also found in all midgut samples. In earlier research, strict acetoclastic methanogens were not acknowledged as constituents of the gut methanogenic communities (Janssen & Kirs, 2008). Furthermore, Methanosarcinaceae members are usually not the predominant microorganisms, but they are frequently found in the guts of insects and ruminants (Janssen & Kirs, 2008). However, recent literature has emerged with contradictory findings about the gut community of *P. marginata* larvae, reporting the presence of *Methanothrix* in the gut of *P. marginata* for the first time (Schroeder et al., 2022).

Furthermore, methanogens abundant in the soil were affiliated with *Methanothrix* and *Methanosarcina*. The soil microbial community has been described as exerting an influence on the gut microbiome of soil organisms such as earthworms (Depkat‐Jakob et al., 2012), and methane emission originates from methanogens that have been ingested. Hence, soil has an effect on the midgut section of the *P. marginata* larva, and *Methanothrix* is a transient member of the methanogenic community inhabiting the midgut. However, a strong selection in the hindgut happened mainly due to the more strict anoxic conditions that shaped the community composition. Our findings indicated that the composition of bacteria and methanogens in the hindgut was more distinctive than in the midgut.

The data about the eukaryotic community composition in the gut of scarabs is relatively limited (Mason et al., 2019; Mohammed et al., 2018). In our study, dietary changes resulted in a clear difference in gut eukaryotic community composition. However, there was not a significant difference in the compartmentalization. Even though the general groups present within the gut were almost identical, their relative abundance varied slightly. Most of the eukaryotic ASVs were assigned to Dikarya. Intramacronucleata species contribute to soil decomposition processes and nutrient cycles (Islam et al., 2022). Additionally, Chrysophyceae members are commonly found in soil; however, their function in insects is largely unknown.

In termites, the digestion of lignocellulosic materials is a complex sequential process involving the gut symbionts. While host functions appear to be primarily responsible for the digestion activity in the foregut and midgut by secretion of digestive enzymes, the symbionts dominate the digestion in the hindgut (Brune, 2006, 2014). In lower termites, flagellate protists that originated from termites' ancestors are known to be involved in digestion (Brune, 2014). However, in our study, no eukaryotes associated with known functions were detected in the gut systems of *P. marginata* larvae. One hypothesis is that most eukaryotes are transiently present, and no specific symbiosis occurs between the larvae and certain eukaryotic groups. An alternative hypothesis is that the detected eukaryotes have unknown functions that are relevant for *P. marginata* larvae, even possibly involving lignocellulose degradation. Further, this result could also be linked to the primer set used in the study, which targets a wide range of eukaryotes (Fiore‐Donno et al., 2018). The use of universal primers for eukaryotes has limitations in detecting a significant portion of the diversity, particularly favouring ciliates while exhibiting negative selection towards Amoebozoa (Fiore‐Donno et al., 2018).

Our random forest approach using ASV tables as input indicated that the biodiversity shifts observed in the gut (hindgut + midgut), leaf diet, hindgut of straw diet, midgut of straw diet, and soil were sufficiently conserved to determine bioindicators. When using the prokaryotic ASV table alone, we observed a 3% error. Nevertheless, when the input was the prokaryotic and eukaryotic concatenated ASV tables, all samples were classified properly. A similar approach has been used to identify bioindicators in coral reefs in a microbiome manipulation experiment (Rosado et al., 2019), in bioreactors studying the biotransformation of hexachlorocyclohexanes (Lian et al., 2020), and in chain elongation reactors (Liu et al., 2022). Our data indicated that the use of group‐specific primers for different domains (Bacteria, Archaea, and Eukarya) may increase the classification accuracy of different microbial communities based on random forest analyses.

The gut microbiome is quite important for various host functions as well as the adaptation of the host to the surrounding environment because it can rapidly adapt and exchange microorganisms with the environment (Shapira, 2016). The hologenome concept describes the co‐evolution of endosymbionts with their hosts (Rosenberg & Zilber‐Rosenberg, 2018). Some insects undergo distinct lifestyles during their larval, pupa, and adult stages, which often requires the microbiota to persist in the environment longer to enable effective transfer to the next generation (Ozbayram et al., 2020). There are many factors affecting the microbial diversity in the gut of scarabs. Feed and soil are sources of microorganisms for the gut of insects (Mason et al., 2019). As in termites, the community pattern along the gut compartments in the insect larvae is dependent on the stable microenvironmental conditions created by the host such as oxygen concentration, pH, redox potential, etc. (Lampert et al., 2019). However, it is important to note that our experiment was conducted within a spatially limited system. As a result, it is likely that the microbiota of the faeces impacted the microbiota of the soil somewhat more than in a natural setting.

The conversion of lignocellulosic biomass to high‐value chemicals and energy carriers has gained great attention through the interest in biorefinery concepts that substitute petrol‐based production. Since the biodegradation of lignocellulose‐rich components is challenging due to their recalcitrant structures, effective approaches are necessary to solubilize these components. Various insects can feed on a lignocellulose‐rich, nutrient‐poor diet thanks to their unique gut structures and effective microbiomes, so they hold great potential as models for biorefineries (Ozbayram et al., 2020). Thus, to mimic the larval degradation process and potentially use it technically, it is crucial to understand the gut microbiome and its host thoroughly and how they jointly break down lignocellulose (Rajeswari et al., 2021). Strategic enrichment of lignocellulose‐degrading communities can be one option to obtain specific cultures for biorefinery applications (Schroeder et al., 2022), and a recent study showed that a culture enriched from gut compartments of *P. marginata* larvae is a promising source for biorefineries using lignocellulosic biomass (Schroeder et al., 2022).

This study explored the influence of different lignocellulosic biomass sources on the gut three‐domain microbial community of *P. marginata* larvae. The findings indicated a notable variation in the microbial community composition among the gut compartments. Although community assembly was not stochastic, the external environmental factors (soil, food type, environmental microbiota) had a bigger influence on the midgut microbiota, while hindgut microbiota was more host‐specific along with the holobiont concept.

## AUTHOR CONTRIBUTIONS

**Emine Gozde Ozbayram:** Conceptualization; investigation; methodology; data curation; formal analysis; validation; visualization; writing – review and editing; writing – original draft. **Sabine Kleinsteuber:** Investigation; methodology; data curation; formal analysis; writing – review and editing. **Heike Sträuber:** Investigation; methodology; writing – review and editing. **Bruna Grosch Schroeder:** Investigation; writing – review and editing. **Ulisses Nunes da Rocha:** Data curation; formal analysis; validation; visualization. **Felipe Borim Corrêa:** Data curation; formal analysis; writing – review and editing. **Hauke Harms:** Writing – review and editing; formal analysis; resources. **Marcell Nikolausz:** Conceptualization; investigation; methodology; formal analysis; writing – review and editing; data curation; supervision; project administration; writing – original draft.

## CONFLICT OF INTEREST STATEMENT

The authors declare no conflict of interest.

## Supporting information


Appendix S1.

## Data Availability

The sequencing data are deposited in the EMBL‐EBI database with the accession number PRJEB72013.
