# Supplementary material for: Three‐domain microbial communities in the gut of Pachnoda marginata larvae: A comparative study revealing opposing trends in gut compartments
Source: Environ Microbiol Rep. 2024 Aug 14;16(4):e13324. doi: 10.1111/1758-2229.13324 (PMC11324371; doi:10.1111/1758-2229.13324)
Supplement: Supplementary file 1 — Appendix S1. [file EMI4-16-e13324-s001.docx]

**APPENDIX**


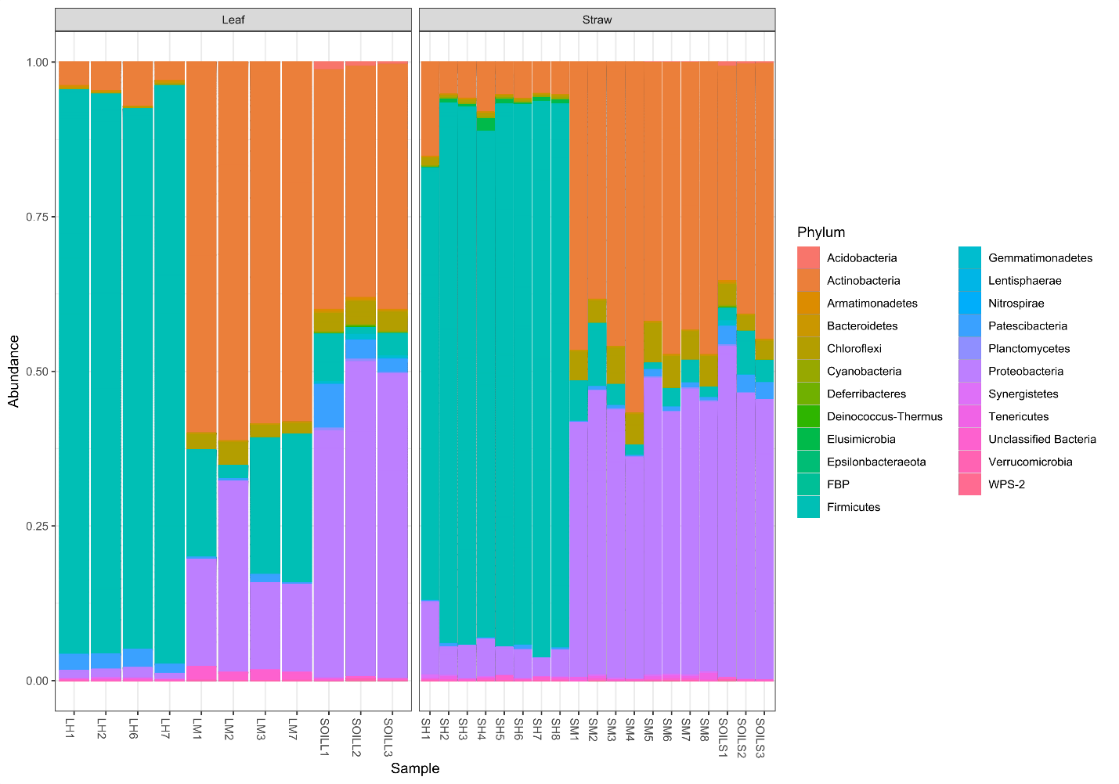


**Figure S1.** Relative abundance of the bacterial phyla within the larval gut compartments (midgut and hindgut) and soil (L: Leaf, S: Straw, H: Hindgut, M: Midgut) .


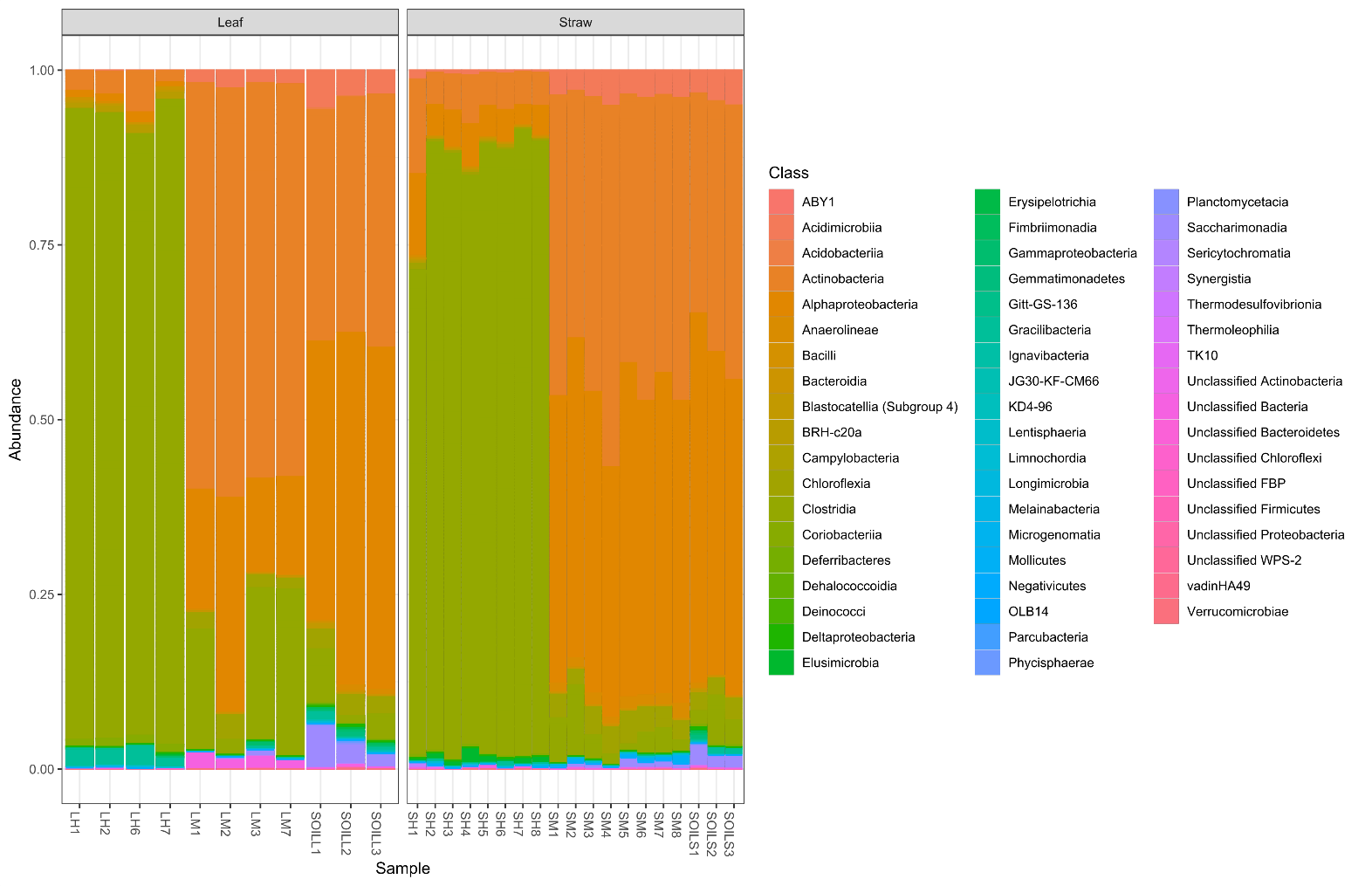


**Figure S2.** Relative abundance of the bacterial classes within the larval gut compartments (midgut and hindgut) and soil (L: Leaf, S: Straw, H: Hindgut, M: Midgut).

**
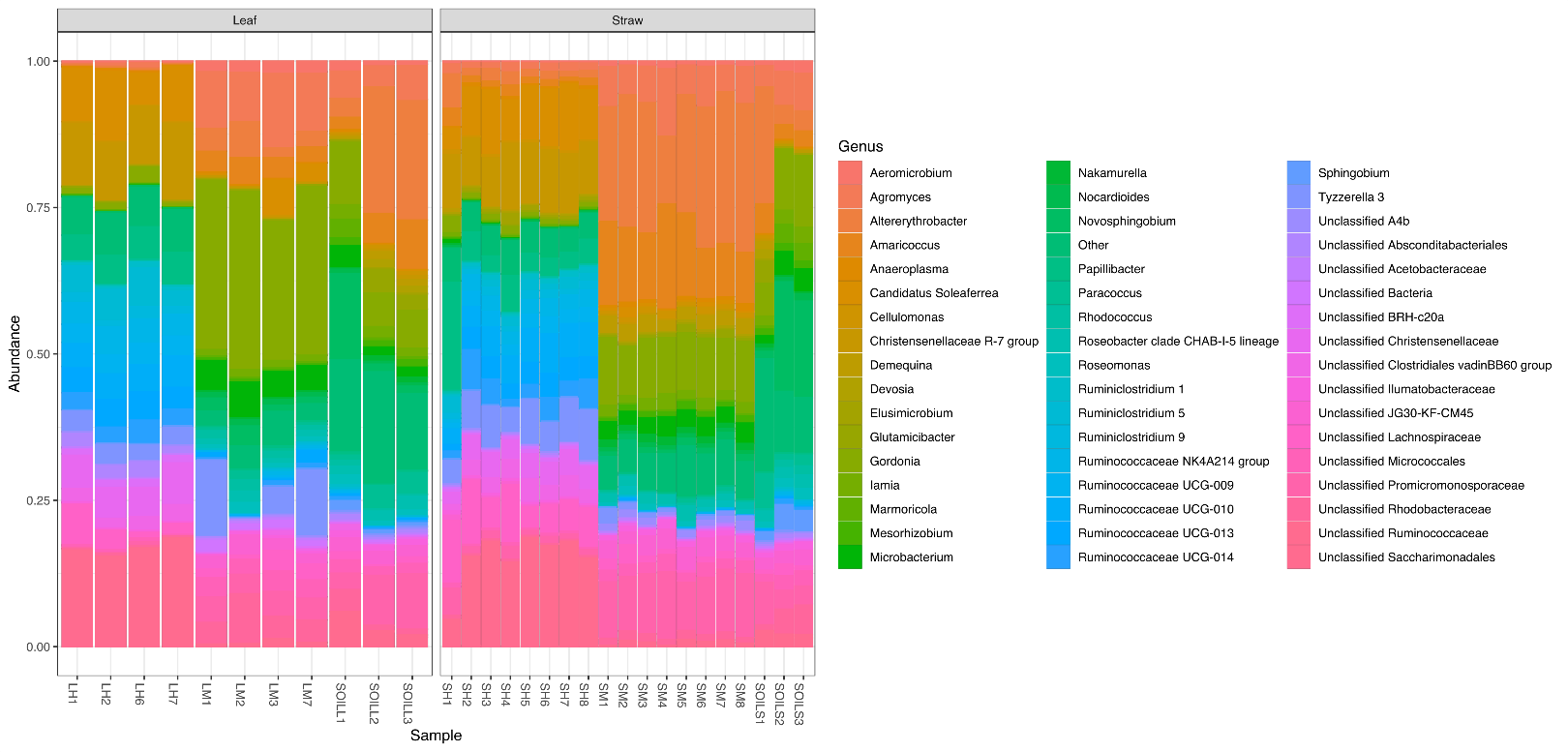
 Figure S3.** The abundance of the 50 most abundant bacterial genera within the larval gut compartments (midgut and hindgut) and soil (L: Leaf, S: Straw, H: Hindgut, M: Midgut).

**
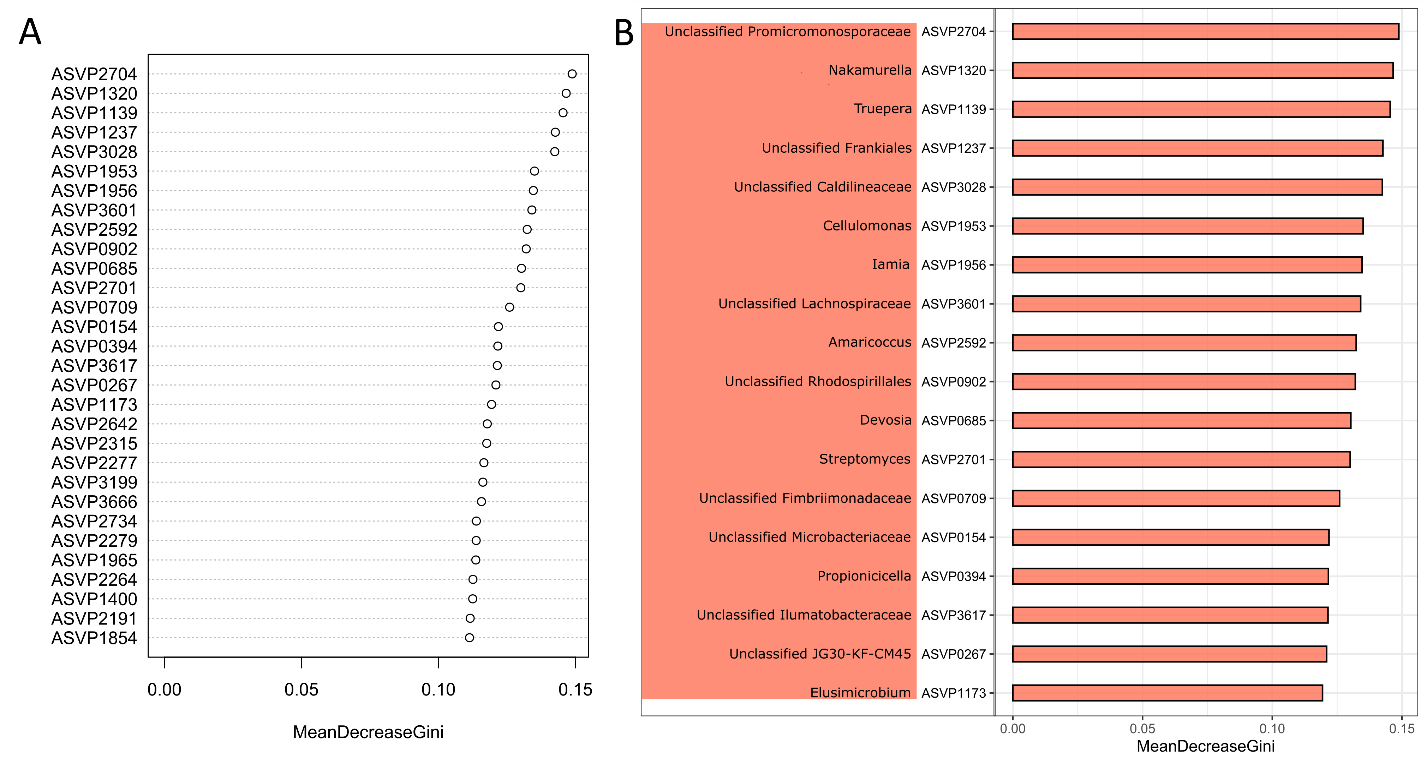
**

**Figure S4.** **A)** Random forest variable importance of 16S ASVs used to classify all selected samples, according to their ASV abundance distribution. **B)** The top-ranked 18 ASVs reducing the uncertainty in the prediction of compartments. According to their ASV abundance distribution, the order of features (from top to bottom) was based on their Mean Decrease Gini scores.

**
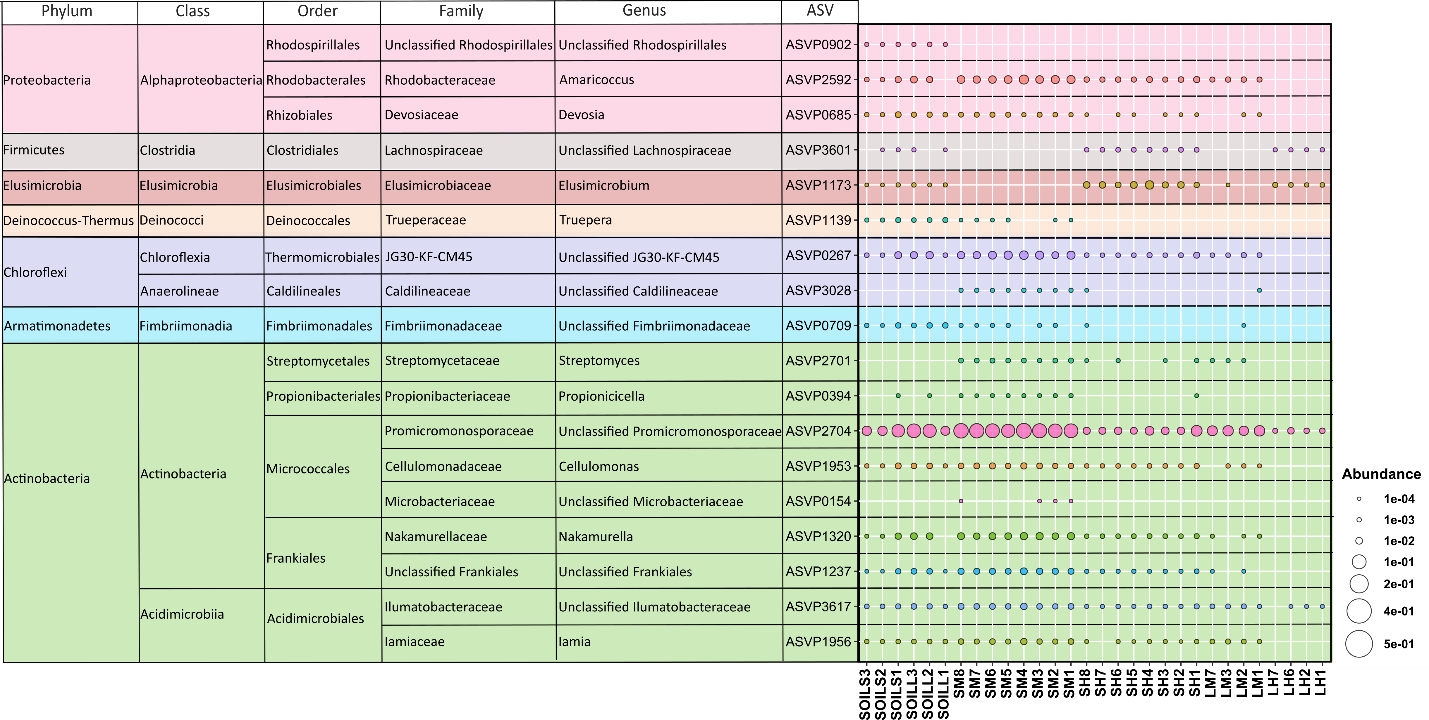
**

**Figure S5.** Relative abundance of the 16S rRNA bioindicator ASVs for indication of larval gut compartments (midgut and hindgut) and soil (L: Leaf, S: Straw, H: Hindgut, M: Midgut).

**
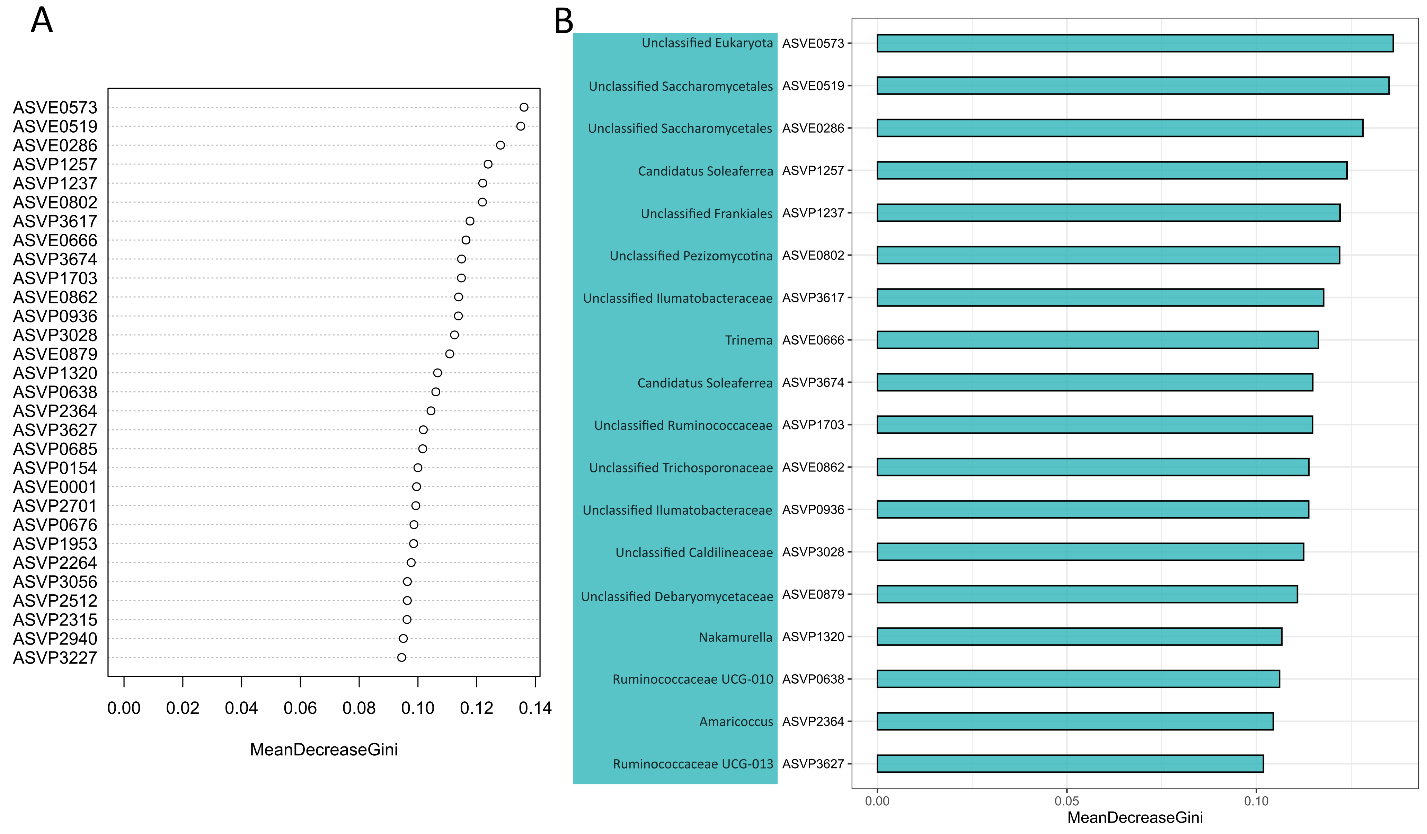
**

**Figure S6.** Random forest variable importance of 16S rRNA and 18S rRNA ASVs used to classify all selected samples, according to their ASV abundance distribution. **B)** The top-ranked 18 ASVs reducing the uncertainty in the prediction of compartments. According to their ASV abundance distribution, the order of features (from top to bottom) was based on their Mean Decrease Gini scores.

**
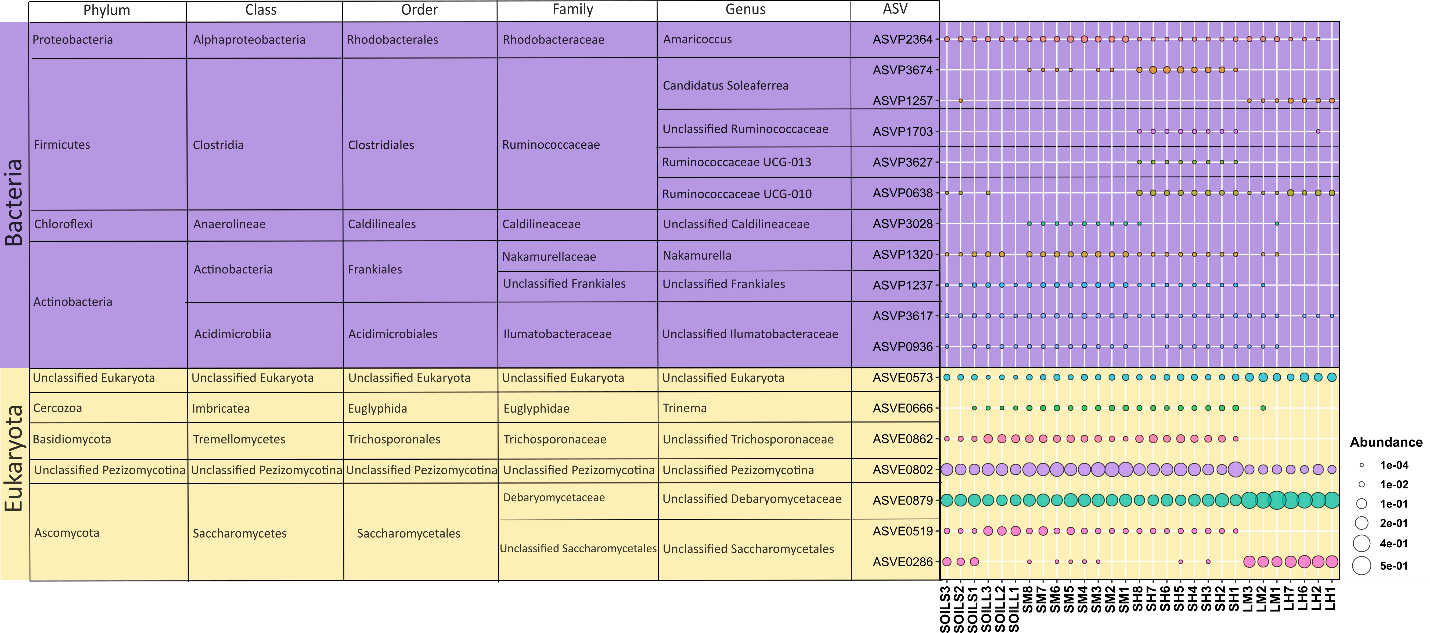
**

**Figure S7.** Relative abundance of the 16S rRNA and 18S rRNA bioindicator ASVs for indication of larval gut compartments (midgut and hindgut) and soil (L: Leaf, S: Straw, H: Hindgut, M: Midgut).

**Table S1.** Confusion matrices were generated using a random forest algorithm (see Exerimental Procedures) for the classification of the samples.

| **Classification** | **Confusion Matrix** | Leaf Gut | Soil | Straw Hindgut | Straw Midgut | **Classification Error** |
| --- | --- | --- | --- | --- | --- | --- |
| Prokaryotes | Leaf Gut | 8 |  |  |  | 0.000 |
|  | Soil |  | 6 |  |  | 0.000 |
|  | Straw Hindgut | 1 |  | 7 |  | 0.125 |
|  | Straw Midgut |  |  |  | 8 | 0.000 |
| Prokaryotes+Fungi | Leaf Gut | 8 |  |  |  | 0.000 |
|  | Soil |  | 6 |  |  | 0.000 |
|  | Straw Hindgut |  |  | 8 |  | 0.000 |
|  | Straw Midgut |  |  |  | 8 | 0.000 |
